# Oral Flea Preventive to Control *Rickettsia typhi*–Infected Fleas on Reservoir Opossums, Galveston, Texas, USA, 2023–2024

**DOI:** 10.3201/eid3106.241817

**Published:** 2025-06

**Authors:** Lucas S. Blanton, Alejandro Villasante-Tezanos

**Affiliations:** University of Texas Medical Branch, Galveston, Texas, USA

**Keywords:** *Rickettsia typhi*, murine typhus, bacteria, rickettsia, vector-borne infections, opossums, fleas, vector control, endemic typhus, flea-borne typhus, Texas, United States

## Abstract

Murine typhus, a fleaborne bacterial disease caused by *Rickettsia typhi*, has reemerged and spread in the United States. We tested spinosad, an oral flea preventive, in opossum flea reservoirs. Spinosad killed 98% of fleas infesting opossums. Oral preventives could control fleas in host species and curb murine typhus spread to humans.

*Rickettsia typhi* is a fleaborne bacterium that causes murine typhus, an acute undifferentiated febrile illness, in humans (*1*). The organism is classically maintained by rats and transmitted by their fleas (*Xenopsylla cheopis*). In the United States, the incidence of murine typhus drastically fell after campaigns to control rat fleas with DDT (*2*). In the past decade, murine typhus has reemerged in the United States and alarmingly increased in incidence and geographic distribution (*3*). In endemic areas of North America, murine typhus has been associated with a transmission cycle involving opossums (*Didelphis virginiana*) and cat fleas (*Ctenocephalides felis*) (*1*). 

One way to reduce murine typhus is field-based control of fleas that transmit *R. typhi* to humans. Spinosad (Comfortis; Elanco, https://farmanimal.elanco.com) is an orally ingested medication (comprising spinosyn A and spinosyn D) approved for use in dogs and cats. A single monthly dose is highly effective in killing fleas by activating nicotinic acetylcholine receptors, leading to flea paralysis and death (*4*). We tested effectiveness of spinosad for killing fleas on opossums in a murine typhus–endemic area. The study was approved by the University of Texas Medical Branch Institutional Animal Care and Use Committee (protocol no. 2104030) and Texas Parks and Wildlife (scientific permit no. SPR-1020154).

## The Study

To capture opossums, we deployed Havahart 1-door cage traps (81 cm × 31 cm × 25 cm) (Woodstream Corporation, https://www.woodstream.com) in Galveston, Texas, USA, during evening hours. We conducted 2 arms, comprising an experimental group and a control group. In the experimental group, we baited traps with canned cat food mixed with a crushed 270-mg spinosad tablet. In the control group, we trapped opossums using cat food without spinosad in the bait. 

Spinosad starts to kill fleas within 30 minutes, and studies show it can kill 100% of fleas within 4 hours of ingestion in dogs and within 24 hours in cats (*4*). To collect dead fleas fallen from opossums, we placed a white terrycloth towel (160 cm × 84 cm) under the trap (Figure; Video). To ensure trapped opossums ingested bait at least 2 hours before flea collection, we checked traps between 4 am and 6 am the morning after setting.

**Figure:**
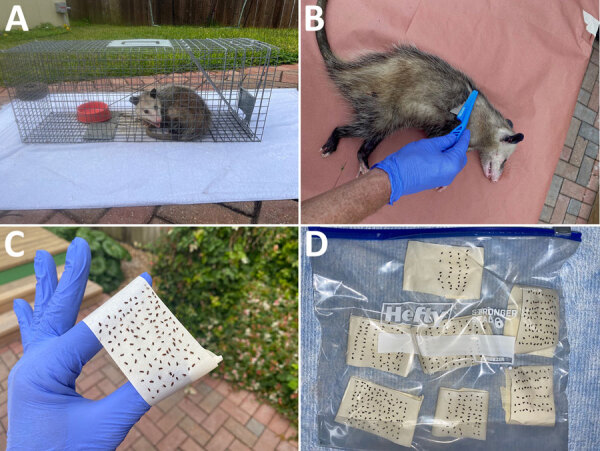
Trapping and flea collection in a study of use of oral flea preventive to control *Rickettsia typhi*–infected fleas on reservoir opossums, Galveston, Texas, USA, 2023–2024. A) Trapped opossum; B) anesthetized opossum being combed for fleas; C) masking tape displaying collected fleas; D) sealable plastic bag containing fleas from a single opossum. To determine the effectiveness of spinosad (Elanco, https://farmanimal.elanco.com) as a flea-killing agent on opossums, cage traps were baited with canned cat food mixed with a crushed 270-mg spinosad flea control tablet for the experimental group and cat food without flea control for opossums in the control group. Fleas collected from opossums ingesting spinosad were almost uniformly dead; thus, they were straightforward to collect, easy to organize when placed on tape, and remained affixed to the tape (panels C, D). On the contrary, fleas in the control group were uniformly alive when collected, moved vigorously, and could not be affixed to the tape in an organized row-by-row manner, as seen for fleas in the experimental group.

**Video:** Video of opossum trapping in a study of use of oral flea preventive to control *Rickettsia typhi*–infected fleas on reservoir opossums, Galveston, Texas, USA, 2023–2024. A cage trap is set over a white towel and baited with canned cat food mixed with a crushed 270-mg spinosad flea control tablet (Elanco, https://farmanimal.elanco.com). As the opossum enters the trap and walks toward the bowl of spinosad-impregnated food, it steps on the trap’s trigger plate, which actuates the closing of the trap door. In this particular trapping, the first of these experiments, the food bowl is almost tipped over by the startled opossum. Subsequent experiments used a commercially available cat food bowl to avoid easily tipping over the food contents.

We used intramuscular ketamine (30 mg/kg) to anesthetize trapped opossums. After adequate sedation, we collected fleas by combing through the fur with a flea comb (Hartz, https://www.hartz.com) for 15 minutes, then placed fleas on masking tape and secured in sealable plastic storage bags (Figure, panels B–D). After we collected fleas from the anesthetized opossum, we collected fleas from the towel. During collections, we observed flea activity and considered moving fleas to be alive and fleas without movement to be dead. All opossums recovered uneventfully from anesthesia and were released at a distant site to avoid subsequent trapping of the same animal. We caught 3 off-target species (1 raccoon and 2 cats) and released those animals on-site without intervening procedures. 

We captured 9 opossums over 19 trap nights: 5 in the experimental group and 4 in control group (Table 1). Six (66.7%) were female and 3 (33.3%) were male. The mean weight was 2.1 kg. All opossums completely ingested food within the trap. We observed fleas on all 9 opossums. Among the opossums that ingested spinosad, fleas were largely immobile and easily combed from fur; fleas were also numerous on the towel and were easily collected and bagged (Figure, panels C, D). In contrast, fleas in the control group moved rapidly within the opossums’ fur; were difficult to comb off, often jumping off the comb; and would frequently escape from the tape after being bagged. 

**Table 1:** Summary of fleas collected from opossums in a study of use of oral flea preventive to control *Rickettsia typhi*–infected fleas on reservoir opossums, Galveston, Texas, USA, 2023–2024*

Opossum no.	Group	Total no. fleas	No. live fleas	No. dead fleas	Proportion dead fleas
1	Experimental	87	7	80	0.920
3	Experimental	8	0	8	1
5	Experimental	715	1	714	0.999
6	Experimental	595	3	592	0.995
8	Experimental	247	2	245	0.992
2	Control	5	5	0	0
4	Control	54	54	0	0
7	Control	89	89	0	0
9	Control	247	247	0	0
Total		2,047	408	1,639	

*Opossums in the experimental group were captured by using traps baited with canned cat food mixed with a crushed 270-mg spinosad flea control tablet (Elanco, https://farmanimal.elanco.com); opossums in the control group were trapped in the same manner but did not receive spinosad. For both groups, opossums were sedated and combed for fleas, which we determined to be alive (moving) or dead (no movement).

We examined collected fleas with a dissecting microscope and identified flea species by taxonomic key (*5*). We pooled fleas (dead and alive) in groups of 5–20 fleas based on the animal from which they were collected. We homogenized a subset of pools from each opossum and extracted DNA, as previously described (*6*). We used a multiplex real-time PCR to amplify and identify *R. typhi* and *R. felis* from flea homogenates (*7*).

We compared the proportion of dead fleas from the experimental and control groups. Because those proportions were bounded by 0 and 1, we transformed proportions by taking the arcsine of their square root (*8*). We needed sample sizes of at least 3 opossums in the experimental and 2 in the control groups to achieve 82% power to reject the null hypothesis with a significance level (α) of 0.05 using a 2-sided 2-sample equal-variance *t*-test. We determined rates of *R. typhi* and *R. felis* flea infection by using previously described methods (*9*) (Appendix).

We collected a total of 2,047 fleas and identified all as *Ct. felis*. The proportion of dead fleas from opossums in the experimental group was 0.98 (range 0.92–1) compared with 0 (p<0.001) in the control group (Table 1). 

We tested 71 pools (comprising 744 fleas) by PCR; 11 pools demonstrated *R. typhi* DNA, and 23 pools demonstrated *R. felis* DNA (Table 2). Those findings correspond to an infection rate of 1.3% (95% CI 0.7–2.2) for *R. typhi* and 3.2% (95% CI 2.2–7.9) for *R. felis*. Sequenced PCR products of portions of rickettsial *htrA* (GenBank accession no. PQ625781) and *sca5* (GenBank accession no. PQ625780) of an *R. typhi*–infected flea pool detected by real-time PCR confirmed 100% homology with *R. typhi* Wilmington type strain.

**Table 2:** Summary of pooled fleas tested in a study of use of oral flea preventive to control *Rickettsia typhi*–infected fleas on reservoir opossums, Galveston, Texas, USA, 2023–2024*

Opossum no.	Total no. fleas	No. pools tested	No. positive pools			Infection rate, % (95% CI)	
			*R. typhi*	*R. felis*		*R. typhi*	*R. felis*
1	50	10	0	2		0 (0–6.3)	4.2 (0.8–13.3)
2	0†	0	NA	NA		NA	NA
3	8	3	0	0		0 (0–25.3)	0 (0–25.3)
4	54	12	9	3		24.2 (12.9–42.7)	6.1 (1.6–16.0)
5	150	10	1	6		0.7 (0–3.3)	5.5 (2.3–12.0)
6	100	10	1	2		1.0 (0.1–4.9)	2.1 (0.4–6.9)
7	89	7	0	2		0 (0–3.3)	2.4 (0.5–7.8)
8	150	10	0	4		0 (0–2.1)	3.2 (1.1–7.8)
9	143	9	0	4		0 (0–2.2)	3.2 (1.1–7.9)
Total	744	71	11	23		1.3 (0.7–2.2)	3.2 (2.2–4.6)

*Flea pools consisted of 5–20 fleas and were tested by duplex real-time PCR for *Rickettsia typhi* and *Rickettsia felis*. NA, not applicable †Five fleas were found within the fur of opossum 2, but fleas escaped and were not collected.

## Conclusions

In this study, we showed that commercially available spinosad (approximately $20 per tablet) effectively kills fleas infesting opossums. The observed animals readily fed on bait laced with the medication, and almost 100% of collected fleas were found dead at least 2 hours after opossums ingested the preventive. Although spinosad caused mild side effects (e.g., vomiting) in dogs and cats in controlled studies (*4*), little data on adverse effects in other species are available. Although we noticed no ill effects in opossums in this study, the sample size was small. 

Consistent with other areas of the United States where murine typhus is endemic, we detected *R. typhi* in cat fleas collected from opossums (*6*,*10*). In contrast, other reports show few fleas collected from domestic cats are infected with *R. typhi (**11*,*12**)*. 

Targeted efforts to control rat fleas via application of DDT on rat runs and harborages during the 1940s and 1950s exemplify how vector control can break the cycle of vectorborne disease transmission. In 1944, murine typhus peaked at 5,401 reported cases, but that number fell to <100 reported cases annually by the mid-1950s after introduction of DDT (*2*,*3*). However, the effects of DDT seemed to have little spillover into opossums (*13*), and the lingering low-level endemicity of murine typhus in the United States was eventually attributed to an alternate *R. typhi* transmission cycle involving opossums and cat fleas (*1*,*3*). Those findings support the need to focus flea control efforts on opossums in murine typhus endemic areas of the United States. More research is required to investigate the proposed methods’ feasibility, effectiveness in nonexperimental conditions, logistics and toxicity among nontarget species, and effects on disease transmission in murine typhus endemic areas. For instance, in high-burden disease locations, field-deployable methods could include manual dispersion of spinosad-impregnated food pellets or timed-release dispenser stations placed at strategic distances.

In conclusion, the growing distribution and incidence of murine typhus in the United States are a call to develop and implement integrated pest control strategies to aid public health prevention (*14*,*15*). We found that oral flea preventive effectively controlled *R. typhi* flea vectors in reservoir opossums. The proposed method could be used to control vector fleas among reservoir hosts and reduce risk for murine typhus in humans.

Appendix Additional information on oral flea preventive to control *Rickettsia typhi*–infected fleas on reservoir opossums, Galveston, Texas, USA, 2023–2024.
