# Supplementary figures and images for: Oral Flea Preventive to Control Rickettsia typhi–Infected Fleas on Reservoir Opossums, Galveston, Texas, USA, 2023–2024
Source: Emerg Infect Dis. 2025 Jun;31(6):1193–6. doi: 10.3201/eid3106.241817 (PMC12123924; doi:10.3201/eid3106.241817)

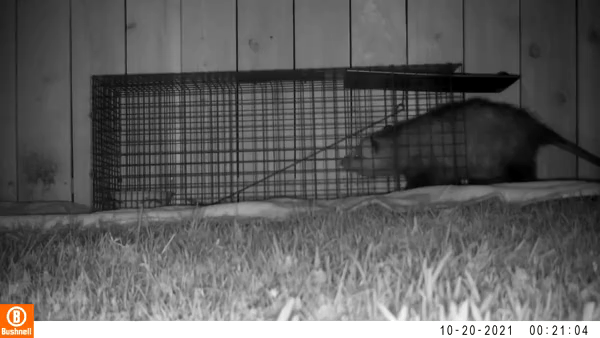

Supplement: Supplementary file 1 [file 24-1817-V.gif]
