# Supplementary material for: Oral Flea Preventive to Control Rickettsia typhi–Infected Fleas on Reservoir Opossums, Galveston, Texas, USA, 2023–2024
Source: Emerg Infect Dis. 2025 Jun;31(6):1193–6. doi: 10.3201/eid3106.241817 (PMC12123924; doi:10.3201/eid3106.241817)
Supplement: Appendix — Additional information on oral flea preventive to control Rickettsia typhi–infected fleas on reservoir opossums, Galveston, Texas, USA, 2023–2024. [file 24-1817-Techapp-s1.pdf]

Article DOI: https://doi.org/10.3201/eid3106.241817

EID cannot ensure accessibility for supplementary materials supplied by authors. Readers who have difficulty accessing supplementary content should contact the authors for assistance.

Oral Flea Preventive to Control *Rickettsia typhi*–Infected Fleas on Reservoir Opossums, Galveston, Texas, USA, 2023–2024

Appendix

Methods

Rationale for Animal Numbers

Sample sizes of at least 3 opossums in the experimental group and 2 opossums in the control group, were determined to be needed to achieve 82% power to reject the null hypothesis with a significance level (α) of 0.05 using a two-sided two-sample equal-variance t-test. To account for the possibility that an animal would not have fleas or technical/logistical problems prevented the collection and counting of fleas, an additional 2 animals in each group were approved by the UTMB Institutional Animal Care and Use Committee.

Additional Trapping Details

Havahart one-door cage traps (81 cm X 31 cm X 25 cm) (Woodstream Corporation, Lancaster, PA, USA) were set in one of three yards in Galveston, Texas. Traps were set on evenings convenient to the principal investigator's schedule and when there was no forecasted precipitation. In the experimental group, traps were baited with ≈3 tablespoons of beef and liver flavored canned cat food mixed with a crushed 270 mg tablet of spinosad (Elanco, Auckland, New Zealand). A control group consisted of opossums trapped using beef and liver flavored canned cat food without the addition of spinosad.

Rationale for Spinosad Dosing

Spinosad is dosed based on weight. It is formulated in several doses (140 mg, 270 mg, 560 mg) for a range of cat and dog weights. There are additional doses available for use in heavier dogs (810 mg and 1,620 mg). The 270 mg dose is approved for use in cats weighing 2.8 to 5.4 kg, which corresponds to the average adult cat. Adult opossums are similar in size to adult cats. We therefore chose to use the 270 mg dose.

Release of Trapped Opossums

After combing for fleas, animals were placed back in their trap, and the traps were placed in a shaded area while the opossums recovered from anesthesia. Once fully recovered, the opossums were released on Pelican Island (a separate island connected to Galveston by a drawbridge) to avoid subsequent trapping of the same animal

Flea Processing and DNA Preparation

Fleas were pooled in 5–20 fleas (organized based on the animal from which they were collected). A subset of flea pools from each opossum was further processed for PCR analysis. Pooled fleas were placed in 2-mL microcentrifuge tubes. The fleas were homogenized using a pair of 4-mm stainless steel ball bearings and 100 µl of molecular grade water in a Qiagen TissueLyser II bead mill (Qiagen, Germantown, MD, USA). Tubes were shaken in the bead mill at 30 Hz for 1 minute. DNA was extracted from flea homogenates using the Qiagen DNeasy Blood and Tissue Kit (Qiagen).

Real-time PCR

To test collected fleas for infection with *R. typhi* or *R. felis*, DNA from flea pools was subjected to a duplex real-time PCR assay to detect *gltA* using primers, probes, and thermocycling conditions as previously described (*1*). Each reaction consisted of 2 uL of template DNA. Positive controls included DNA from cell culture stocks of *R. typhi* (Galveston strain) and *R. felis* (Pedreira strain). Molecular-grade water was used as a negative control. All samples were run in duplicate.

Conventional PCR

To confirm the presence of *R. typhi* by sequencing, DNA from an *R. typhi*-infected flea pool (detected by the above-mentioned real-time PCR assay), was subjected to conventional PCR to detect portions of rickettsial *sca5* and *htrA*. Primers for *sca5* included 120–2788 and

120–3599 with thermocycling conditions as described elsewhere (*2*). Primers and thermocycling conditions to amplify *htrA* were used as described elsewhere (*3*). Reactions included 5 uL of template DNA. Positive controls for *sca5* and *htrA* PCR runs included DNA from a cell culture stock of *R. sibirica,* and negative controls consisted of molecular-grade water.

Sequencing

Conventional PCR products were processed for sequencing with the QIAquick PCR Purification Kit (Qiagen). These PCR products were sent to the UTMB Molecular Genomic Core Laboratory for bidirectional Sanger sequencing. Sequencing was performed with a Thermo Fisher SeqStudio Flex 24 instrument (Waltham, MA, USA) using the aforementioned forward and reverse *sca5* and *htrA* primers.

Sequence Analysis

Analysis of sequences was performed using the Lasergene 17 software suite (DNASTAR, Madison, WI, USA). The bidirectional sequences were assembled using the software's SeqMan Ultra application. After assembly, chromatograms were inspected to ensure there was consensus between the two sequences. The end sequences, not overlapping with the corresponding sequence generated from the opposite direction, were trimmed. Using the National Center for Biotechnology Information (NCBI) nucleotide BLAST search, sequences were queried for database match. Accession numbers for these sequences were then generated through GenBank, the NCBI's public sequence repository.

References

1. Karpathy SE, Hayes EK, Williams AM, Hu R, Krueger L, Bennett S, et al. Detection of *Rickettsia felis* and *Rickettsia typhi* in an area of California endemic for murine typhus. Clin Microbiol Infect. 2009;15(Suppl 2):218–9. PubMed https://doi.org/10.1111/j.1469-0691.2008.02140.x

2. Roux V, Raoult D. Phylogenetic analysis of members of the genus *Rickettsia* using the gene encoding the outer-membrane protein rOmpB (ompB). Int J Syst Evol Microbiol. 2000;50:1449–55. PubMed https://doi.org/10.1099/00207713-50-4-1449

3. Webb L, Carl M, Malloy DC, Dasch GA, Azad AF. Detection of murine typhus infection in fleas by using the polymerase chain reaction. J Clin Microbiol. 1990;28:530–4. PubMed https://doi.org/10.1128/jcm.28.3.530-534.1990
